# RECURRENT HERPES ZOSTER DUPLEX SYMMETRICUS IN HIV INFECTION

**DOI:** 10.4103/0019-5154.39741

**Published:** 2008

**Authors:** T S Rajashekar, Gurcharan Singh, V Shivakumar, Rajendra Okade

**Affiliations:** *From Department of Dermatology, Sri Devaraj Urs Medical College, Tamaka, Kolar - 563 101, Karnataka, India*

**Keywords:** *Contralateral side recurrent zoster*, *same dermatome*

## Abstract

A HIV infected patirent with recurrent herpes zoster has been presented. Recurrence of herpes zoster contralaterally on the same dermatome and concomitant occurrence of three viral infections, viz. HIV, HPV and VZV in the same patient were the unusual and interesting observations in the present case report.

## Introduction

Herpes zoster is a neuroectodermal viral infection which afflicts one or more closely grouped, spinal or cranial nerves, resulting in unilateral radicular pain and vesicular eruption limited to a dermatome innervated by that nerve.^1^ In immunocompromised individuals zoster may involve more dermatomes or it may be recurrent within the same dermatome or may be associated with generalized dissemination.^2^ Noncontiguous multidermatomal herpes zoster is very rare in both immunocompetent and immunocompromised persons. Most of the reported cases have been limited to two noncontiguous dermatomes.^3^ We report a very rare and interesting clinical presentation of herpes zoster recurring on the contralateral side of the same dermatome in an immunocompromised individual.

## Case Report

A 30-year-old male patient presented with multiple, painful, fluid-filled skin lesions of eight days duration with history of similar lesions on the opposite flank one and a half years back. There was history of multiple unprotected sexual exposures with commercial sex workers.

Examination revealed localized, multiple, grouped vesicles, erosions, ulcers over erythematous base with oozing and crusting over the right side of the abdomen corresponding to T-10 dermatome. Few isolated vesicles were seen over the chest and upper back. Multiple hypopigmented/skin-colored plaques with scarring were present over T-10 dermatome on the left side, corresponding to previous episode of similar skin lesions.

A solitary verrucous papule was present over the shaft of the penis. Other mucosae were normal.

Bilateral inguinal and right axillary lymph nodes were enlarged, discrete, firm and freely mobile. There was no clinical evidence of internal malignancy.

A clinical diagnosis of recurrent herpes zoster with genital condyloma accuminata was made.

Immunocompromised state was suspected and on investigation, ELISA for HIV-1 was positive. Cytodiagnosis (Tzanck smear) was consistent with herpes zoster.

## Discussion

Herpes zoster results in a vesicular rash, which is unilateral in most cases and usually involves thoracic dermatomes, a feature which is of diagnostic value clinically.^4^

Though typically unilateral, multiple dermatomal involvement and bilateral asymmetrical distribution of herpes zoster lesions have been reported. This presentation has been referred to as zoster duplex unilateralis or bilateralis depending on whether one or both halves of the body are involved.^3^,^4^ There are existing case reports of simultaneous trigeminal and lumbar involvement^5^, asymmetrical trigeminal and thoracic involvement^4^ and bilaterally symmetrical thoracic involvement.^6^ In the present case involvement of the same dermatome (T-10) has recurred on the contralateral side (duplex symmetricus). One other report exists of herpes zoster involving T-8 to T-10 dermatomes in an immunocompromised female patient with scarring of the same dermatomes on the other half of the body.^7^

Recurrent zoster may develop at the site of previous eruption or at a different site and the interval between the first eruption and recurrence may vary from one week to 30 years.^8^ In our case recurrence occurred one and a half years after initial episode.

One episode of zoster may enhance immune response to the levels that are sufficient to prevent recurrences and cellular immunity plays an important role in limiting the extent of primary infection as well as preventing the reactivation of latent virus.^9^

In immunocompromised individuals like HIV patients, herpes zoster may recur within the same dermatome.^2^,^10^ Patients having malignancy, especially lymphomas, those on cytotoxic or immunosuppressive therapy, apart from infections with human immunodeficiency virus are at risk of repeated and disseminated zoster eruptions due to impaired cellular immunity. Second attack of herpes zoster in immunocompetent individuals, though described, is rare.^11^

Recurrence of herpes zoster contralaterally on the same dermatome and concomitant occurrence of three viral infections, viz. HIV, HPV and VZV in the same patient were the unusual and interesting observations in the present case report.
